# Robot System Assistant (RoSA): evaluation of touch and speech input modalities for on-site HRI and telerobotics

**DOI:** 10.3389/frobt.2025.1561188

**Published:** 2025-07-30

**Authors:** Dominykas Strazdas, Matthias Busch, Rijin Shaji, Ingo Siegert, Ayoub Al-Hamadi

**Affiliations:** ^1^ Neuro-Information Technology Group, Faculty of Electrical Engineering and Information Technology, Otto-von-Guericke-University, Magdeburg, Germany; ^2^ Mobile Dialog Systems, Faculty of Electrical Engineering and Information Technology, Otto-von-Guericke-University, Magdeburg, Germany

**Keywords:** human-robot interaction, telerobotics, touch, speech, multimodal, user study, cobot, remote robotics

## Abstract

Future work scenarios envision increased collaboration between humans and robots, emphasizing the need for versatile interaction modalities. Robotic systems can support various use cases, including on-site operations and telerobotics. This study investigates a hybrid interaction model in which a single user engages with the same robot both on-site and remotely. Specifically, the Robot System Assistant (RoSA) framework is evaluated to assess the effectiveness of touch and speech input modalities in these contexts. The participants interact with two robots, *Rosa* and *Ari*, utilizing both input modalities. The results reveal that touch input excels in precision and task efficiency, while speech input is preferred for its intuitive and natural interaction flow. These findings contribute to understanding the complementary roles of touch and speech in hybrid systems and their potential for future telerobotic applications.

## 1 Introduction

The ongoing development of the industry is driven by new ideas and the necessity for adaptation. Recent advancements in technology, including the emergence of the Internet of Robotic Things (IoRT) (Romeo et al., 2020), improvements in processing capabilities from newer generations of processors, and better load management via cloud and edge computing (Afanasyev et al., 2019), allows for the creation of more advanced robotic applications. The advancements in hardware and software have enabled an effective integration of on-site and remote robotics, leading to increased flexibility and efficiency in industrial operations (Sadiku et al., 2023; Morgan et al., 2022). On-site robotics typically operate within the boundaries of a workplace environment, requiring minimal latency to interact efficiently with the environment, while remote robotics involves controlling robots over a distance through communication technologies (Hentout et al., 2019).

The high degree of robot automation shifts the user’s role towards that of a supervisor, allowing them to oversee and control robotic operations either locally or through telepresence, making telerobotics a crucial component in Human Robot Interaction (HRI) and future developments.

Supervising and directing task-oriented semi-autonomous robots can be done remotely, as long as the delay between the operator’s commands and the robot’s response and task status, is within the time range tolerable by the process. This requires a reliable communication system, as well as an intuitive and user-friendly interface that allows the operator to interact with the robot as naturally as possible, regardless of their physical location (Su et al., 2023; Hempel and Al-Hamadi, 2023) Figure 1 illustrates a system where an operator can control the robot in both local and remote scenarios using the same interaction modalities.

**FIGURE 1:**
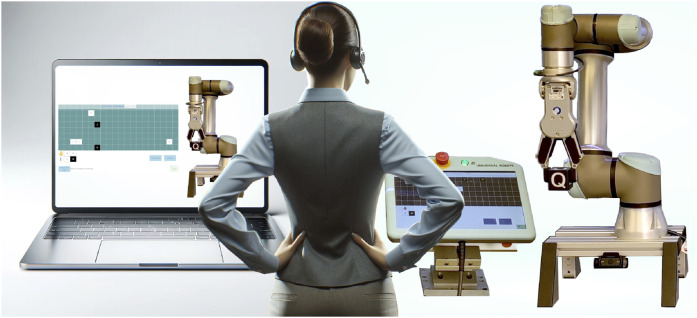
The local interaction on-site and remote interaction is seamless when using the same interaction modalities, getting the same feedback from the robot.

The emergence of collaborative robots, or cobots, has further reduced the barriers between humans and their automated counterparts, enabling new interaction possibilities using the strengths of each (Keshvarparast et al., 2024). This paper will focus on addressing the challenge of integrating intuitive interfaces for human-robot collaboration by developing interaction modalities that can be effectively used for both local and remote environments, thereby overcoming the limitations of current telerobotic systems.

Previous iterations of the Robot System Assistant (RoSA) framework have explored various multimodal interaction techniques to enhance HRI. RoSA 1 laid the foundation by simulating basic interaction capabilities (Strazdas et al., 2020) (as wizard of Oz), while RoSA 2 expanded on this framework by integrating speech and gesture recognition, creating a real operational system (Strazdas et al., 2022).

Building on earlier versions that highlighted issues such as conflicting inputs and the need for better input management strategies, RoSA 3 tries to improve the interaction efficiency and user satisfaction by refining the multimodal system and incorporating new features like touch screen input. This paper evaluates touch and speech input modalities for RoSA, identifying the strengths and weaknesses of each in terms of user efficiency, task accuracy, and satisfaction. The study aims to provide insights into how these modalities can be effectively used in both local and remote settings, thereby contributing to the development of more intuitive and accessible HRI systems.

To further contextualize our research, we review existing approaches in HRI, focusing on collaborative robots, telerobotics, and multimodal interfaces. This section highlights the key findings and challenges faced by current systems.

## 2 Related work

Schmaus et al. (2020) demonstrate that action-level robot control offers significant benefits for teleoperation by executing commands through a local control loop, which enhances robustness against communication delays. This user-friendly and intuitive approach enables novice operators, without robotics or control experience, to perform tasks effectively. While action-level control addresses the challenges posed by latency, task-level authoring, as described by Senft et al. (2021), complements this by allowing operators to define high-level command sequences.

Together, these approaches improve the usability and efficiency of remote control systems, though they do not fully eliminate issues such as complex task planning and environmental adaptation. It should be noted that action-level control alone has limitations. When using the robot to do a sequence of tasks, users must specify each action, wait for it to be executed, and then specify the next one, which may not be optimum. Action-level control, like other high-level control techniques, may also limit the robot to its predefined language.

The usage of web-based platforms for telerobotics is presented in many research studies. In a study by Kapić et al. a web application for remote controlling a Robot Operating System (ROS) robot, using a virtual joystick interface, is presented Kapić et al. (2021). In another study, a web-based platform for remotely controlling robotic manipulators is validated with a real UR3 manipulator (Stefanuto et al., 2023).

These studies highlight the use of backend systems for processing and managing complex ROS tasks, ensuring a streamlined experience for remote users. Both emphasize the importance of real-time communication through WebSockets and 3D visual interfaces, which are crucial for enhancing user interaction and ensuring precise robot control in telerobotic systems.

Current telerobotic systems face several limitations, including high latency, lack of robust multimodal integration, and usability issues Studying and comparing different interaction modalities is essential to address these challenges. Bongiovanni et al. (2022) compared gestural and touchscreen interfaces using a smartwatch and a tablet for controlling a lightweight industrial robot, highlighting that both methods were well-received but required additional hardware for gesture recognition.

Another study compared four modalities—speech, gesture, touchscreen, and a 3D tracking device—for robot programming in small and medium-sized enterprises, using a Wizard of Oz experiment with tasks like pick and place, object assembly, and welding (Profanter et al., 2015). Users preferred touchscreen and gesture over the 3D tracking device, while speech was the least favored due to difficulties in naming unknown objects and specifying precise positions. These studies reveal that while touchscreen and gesture inputs are promising, the lack of seamless integration across modalities and challenges with speech recognition hinder the usability of telerobotic systems.

The speech, gesture, and touchscreen modalities were also studied in the context of the In-Vehicle Infotainment System (IVIS) (Angelini et al., 2016). It turns out users had fewer interactions when using the speech modality, and a shorter task completion time was observed for the touchscreen modality. The study concluded that the speech modality was more suitable for hands-free tasks, while the touchscreen modality was more efficient for precision tasks. These findings are relevant to RoSA, as they provide insights into the strengths and weaknesses of different interaction modalities for cobots in various scenarios.

## 3 System design and architecture

### 3.1 Overview and hardware

**FIGURE 2:**
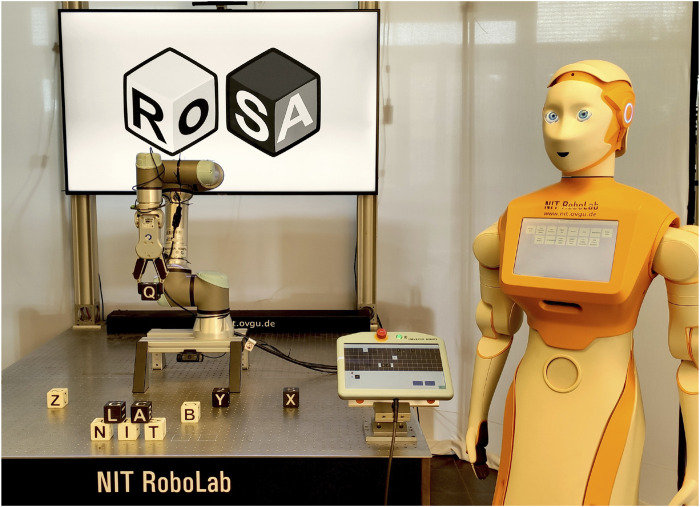
Robots used in the experiment: *Rosa* (stationary, UR5e with a gripper) and *Ari* (humanoid, by PAL Robotics), both capable of speech and touchscreen interaction.

The RoSA system allows users to engage with robots through multiple modalities, such as speech, gestures, touchscreen interface, proximity, and attention detection. The system incorporates two robots: *Rosa*, a stationary industrial UR5e equipped with a gripper used in previous studies, and *Ari*, a humanoid robot from PAL Robotics, which is a new addition to the system. (Note: RoSA refers to the overall system, while *Rosa* denotes the stationary robot.) Both robots are shown in Figure 2.

A projector is utilized to highlight objects and can also exhibit directing feedback, if necessary. Monitors are used to provide visual feedback, whereas loudspeakers are used to deliver auditory feedback, such as speech output or signal tones. The Microsoft Azure Kinect camera captures both color images and depth data using a Time of Flight (ToF) sensor. Additional cameras are used to capture the scene and participants, and the gathered data can later be used for evaluation purposes. The Jabra 930Pro, a wireless headset, is utilized for the purpose of voice recognition.

The stationary robot *Rosa* is securely attached to a robust metal table and is capable of manipulating letter cubes by utilizing the OnRobot RG6 gripper, as used in the previous experiments. The Universal Robot teach pendant, initially intended for direct robot programming, is repurposed as a versatile touchscreen interface that displays a customized Guided User Interface (GUI) explained in the following sections. A camera is positioned close to the gripper to provide *Rosa* with visual access to the surroundings, while a TV monitor in the background provides information and interaction feedback.

The humanoid robot *Ari* possesses a range of characteristics that enable it to engage in basic interactions with people. The robot is outfitted with wheels for locomotion and possesses the capacity to execute basic movements with its head and arms. The robot features a 10-inch touchscreen integrated into its body. In addition, *Ari* perceives its surroundings using a built-in RGB camera and two RGB-D cameras.

### 3.2 Interaction concept

Both robots can align themselves towards the user or go into a “sleep” position if unengaged. To engage a robot, the user must initiate interaction via touch or speech input. At any given time, only one user can be actively engaged with a robot, and only inputs from the engaged user are processed. Although the system supports multi-user interaction and attention tracking, as demonstrated by Abdelrahman et al. (2022), these features were disabled during the experiment. Only one participant was present at a time, and attention detection was turned off. Therefore, no user differentiation or automatic disengagement occurred. Speech input omits positional information and works identically for both robots in local and remote scenarios. Local touch input is robot-specific, while remote touch can switch between robots and mirrors local input.

**FIGURE 3:**
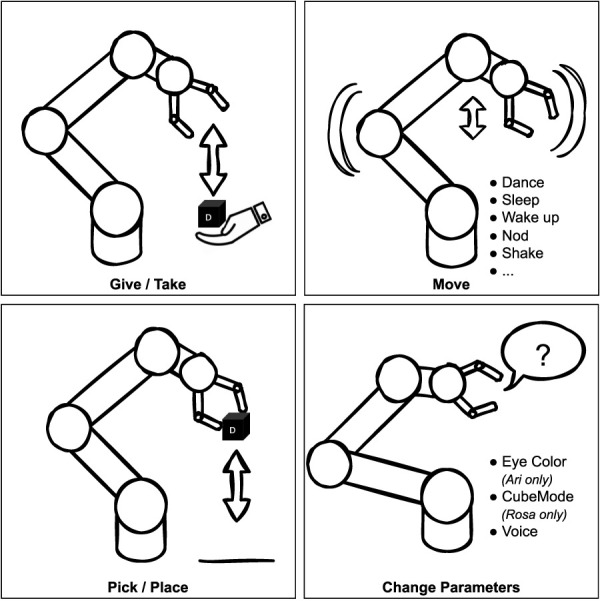
Different possible *RobotActions*.

Both robots can perform actions like greeting, dancing, replaying specific pre-programmed sequences, and allow a level of personalization: voice (both robots) and eye color (*Ari*). Figure 3 summarizes the possible actions the robots can perform.

*Rosa* includes a cube-gripping scenario called CubeMode, enabling letter cube manipulation, which remains consistent with previous studies. Regarding this mode, two essential pieces of information are required to execute a *CubeAction*: a source *CubeMessage* and a destination *CubeMessage*. A *CubeMessage* is the smallest piece of information a user can express in this scenario and is a class that can contain the color, letter, or position of a letter cube. The position can be defined by XY coordinates on the table, the sequential position of a word, or it can be the hand of the user, or the gripper itself. The preceding publication RoSA 2 offers comprehensive information regarding the various *CubeMessage* permutations and their resulting outcomes (Strazdas et al., 2022).

**FIGURE 4:**
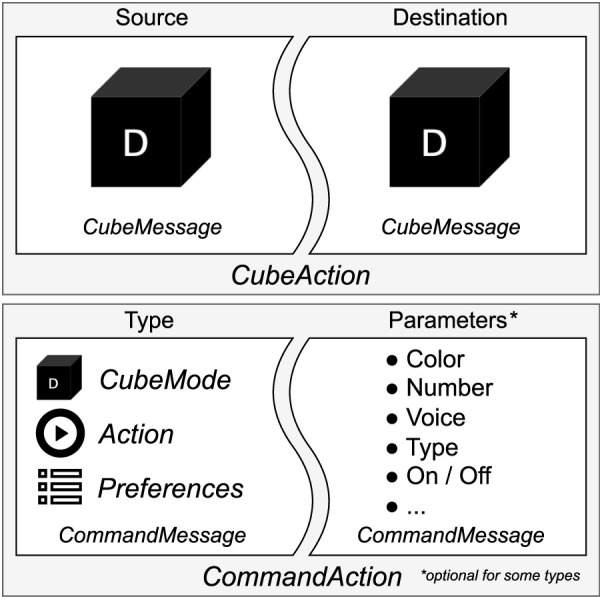
*CommandAction* and *CubeAction*.

For all other interactions besides cubes, *CommandActions* are composed using *CommandMessages* by defining a command type and optionally the necessary command parameters. The splitting of the actions into small information parts allows the system to handle multimodal messages from different inputs. A summary of used message types can be seen in Figure 4.

### 3.3 System architecture

**FIGURE 5:**
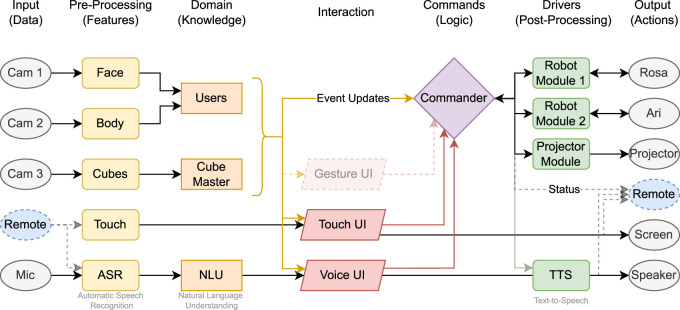
Overview of the system workflow. Sensor data and user input (left) are processed by dedicated ROS 1 (Noetic) nodes. The information is aggregated and resolved by the *Commander*, which decides on the appropriate system action. Outputs are executed via robot modules, the projector, or remote interfaces.

The system overview is presented as a flowchart in Figure 5. Each element represents a separate ROS 1 (Noetic) node responsible for input (data), pre-processing (features), domain (knowledge), different user interface modalities (Touch UI, Voice UI, Gesture UI), and output mechanisms (actions). The main information flow is from left to right, however some system variables (e.g., current robot or cube status) can be received by the according modules immediately on information update (ROS subscription model). These connections are not specifically depicted to preserve the clear direction of information processing.

The system incorporates interactions between components such as Robot Modules, Projector Module, and external and remote devices and the main logic gate called *Commander*.

### 3.4 Commander

**FIGURE 6:**
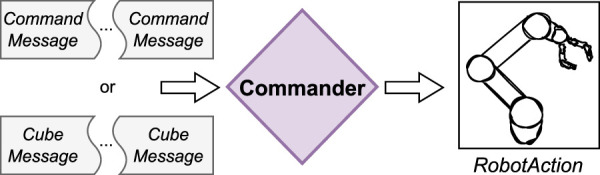
*Commander* assembles the messages and creates a corresponding *RobotAction*.

The RoSA system’s “Commander” module was introduced to support advanced task-oriented, multimodal robot control by managing data from multiple input modalities. Its main goal is to assemble the messages to a corresponding *RobotAction* (see Figure 6). Commander module goes beyond traditional First In First Out (FIFO) paradigms to enable flexible, context-aware interactions by progressively gathering all required information before executing actions. For example, it allows users to specify a destination before selecting a source, leading to more natural commands like “give me that cube” while pointing at it later.

### 3.5 Touch module

The development of the touchscreen interface began with an online UI design survey, followed by a paper prototyping study, and ultimately led to the creation and integration of a React-based web application into the existing system via ROS. The goal was to provide users with an intuitive, accessible interface for robot control, compatible with any web-enabled device, including robot manufacturer-provided monitors.

The implementation utilized React along with ROS libraries like roslibjs and rosbridgesuite to enable seamless communication between the interface and ROS modules. A modular design approach was taken to ensure the application’s independence from other system components, improving flexibility and usability.

**FIGURE 7:**
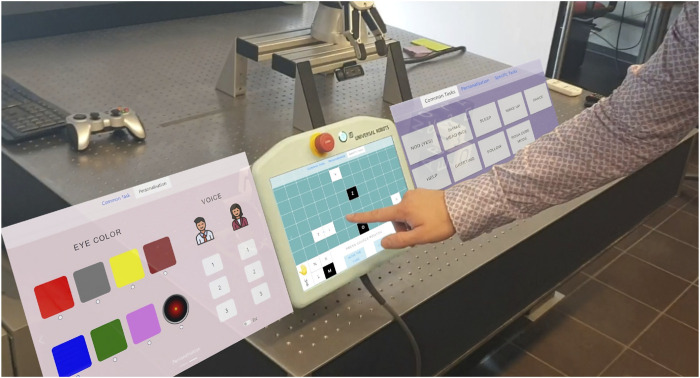
Touchscreen app on *Rosa’s* screen in CubeMode with other available pages (screenshot mockup left and right), which would be available by sliding or tab selection.

The application features three primary pages: *Common Tasks*, *Personalization*, and *Specific Tasks*, designed with a minimal and intuitive UI to reduce complexity. Users can navigate by swiping between pages or using a top tab bar. Figure 7 shows the three pages of the touch interface. The Common Tasks page includes functions that both robots can perform, while the Personalization page allows for voice and language customization, and for *Ari*, eye color changes.

The Specific Tasks page highlights the distinctive functionalities of each robot. For *Rosa*, it activates the CubeMode, displaying a grid that represents the current positions of the cubes. Users can interact with this grid by selecting a cube’s current location (*Source*) and specifying its target location (*Destination*). The interface includes a “Gripper” button, enabling the robot to pick up and hold a cube, as well as a “Hand” button for transferring a cube to or from the user’s hand.

Additional control options include a “Clean Up” button, which commands *Rosa* to return all cubes to their default starting positions, and a “Cancel” button to terminate or reset the current action or selection. Each interaction, such as pressing a button or selecting a cube on the grid, generates a corresponding *CubeMessage* or *CommandMessage*.

As a form of feedback, the application displays UI changes after each interaction, indicating the system’s response and asks the user to wait while the robots are moving, until a stable system status is reached.

### 3.6 Speech module

**FIGURE 8:**
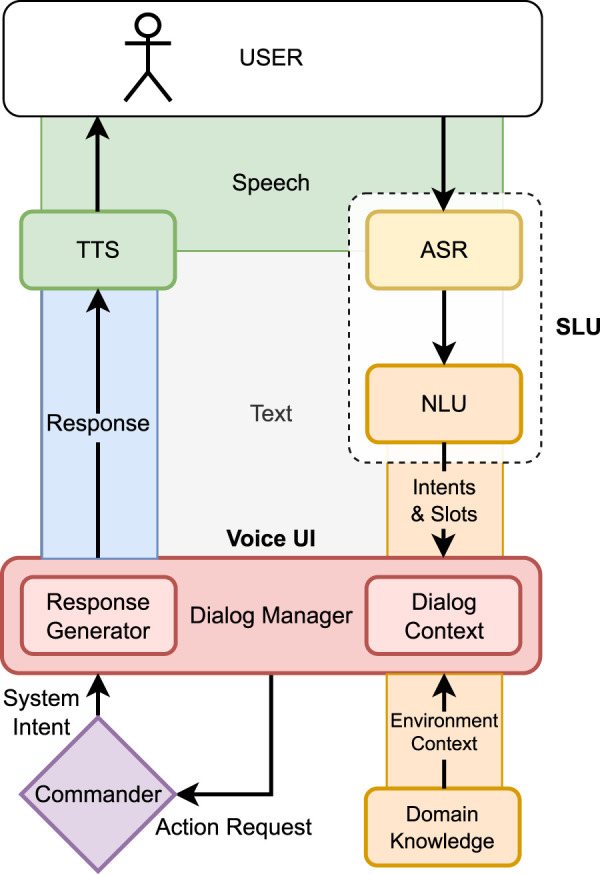
Architecture of the *Voice UI* pipeline.

The architecture of our speech module, as depicted in Figure 8, is modelled after a classical dialogue system processing pipeline approach (McTear, 2022). The pipeline consists of various specialized modules, each performing a distinct task and passing its results to the next module in sequence. While it is conceivable to utilize online services for individual components of the dialogue pipeline, the current requirements demand that it operates locally on the robot with limited resources.

The initial stage involves the acoustic signal being captured by a microphone and subsequently transformed into text by an Automatic Speech Recognition (ASR) component. The Natural Language Understanding (NLU) component seeks to determine the user’s request from their utterances following this conversion.

NLU components are often trained using Natural Language Processing machine learning models. Developers provide an *interaction model*, which consists of intents and a list of training examples (utterances). Intents define commands and instructions that the user might utter during the interaction; slots specify additional parameters for these commands.

Spoken Language Understanding (SLU) components combine ASR and NLU functionalities in a single unit for certain use cases (Radfar et al., 2021). This means that an ASR component is not necessarily required, as intents are directly derived from the acoustic signal by an end-to-end machine learning module.

However, this can introduce limitations regarding the complexity of intent recognition. As a trade-off, the SLU methods have lower resource consumption at the expense of the complexity of the *interaction model*.

For the RoSA 3 model, we decided to use the SLU technology developed by PicoVoice^1^, which specializes in resource-efficient models capable of directly mapping audio signals onto intents. This integration allows for a more resource-efficient implementation of a Voice User Interface (VUI).

The decision-making process of the VUI is orchestrated by the Dialogue Manager (DM), which is informed by the outcomes of the NLU or SLU components. This process also considers context information representing the state of the conversation and system context. In the RoSA system, the Commander documents information such as currently running actions or error situations. Through the “Users” and “Cube-Master” components that contain the domain knowledge (see flowchart in Figure 5), relevant environmental context can be modeled. Furthermore, the VUI can utilize domain knowledge from the Users and Cube-Master components to validate user inputs. If a valid Cube or Command message cannot be created from the user’s input, the DM will directly ask the user for correction or additional information.

In the context of RoSA 3, the VUI is not the only input modality; therefore, Cube or Command messages for an action are sent to the Commander. If the Commander does not set an error or abort status, the current user request is then considered completed. Additionally, the Commander has the possibility to convey direct system statements or confirmations to the user via Text-to-Speech (TTS). This ensures that the system can draw attention to itself at any time in case of an error. These system responses are available to the DM of the Voice UI component in the system status, in case the user reacts to such a message with “pardon?”, triggering a *Repeat-Intent*.

Regarding the dialogue context, there are two distinct dialogue states: Cube Mode and RoSA Commands. Each state offers help for its respective intents and adjusts the intents accordingly (Activate Cube Mode/Deactivate Cube Mode; Activate “Normal” Mode/Deactivate Normal Mode). Each state is linked to its specific NLU model.

To respond to the user, the Text Generation (TG) component must first formulate a textual response, which is then converted into an audio signal by the TTS component. For RoSA, various synthetic voices are available to generate this audio stream, thus concluding the dialogue turn of the system. The TG component utilizes predefined text blocks. Future methodologies of Natural Language Generation, such as Large Language Models, could enhance this process.

## 4 User study design

### 4.1 Experimental setup

This study is a continuation of previous RoSA studies with the intent of consistency and compatibility for comparison reasons. The inclusion of new features, such as the humanoid robot *Ari* and a touchscreen interface, expanded the previous approach. Feedback from participants in previous studies, such as *“I wish I knew the system was capable of this …”*, motivated the introduction of a learning phase. During this phase, users complete a guided tutorial with specific tasks, ensuring they understand and utilize each interaction modality at least once. Tasks are considered complete only after successful execution using the designated modality.

Following the learning phase, an exploration phase allows participants to freely interact with the system while still having a task in the background. In this phase, no specific instructions are provided on how to accomplish the tasks, encouraging users to choose their preferred modalities. This procedure is repeated across both robots and modalities, with participants periodically returning to the monitor for task updates.

Throughout the experiment, multiple cameras capture participant interactions from various angles. Behind a partition, a supervisor monitors the session, taking notes and using a keyboard with predefined hotkeys to log noteworthy events. These events include incorrect robot responses, unresponsiveness to speech commands, misunderstandings of speech inputs, system errors, or issues such as the gripper dropping a cube. The system’s activity logs are later merged with questionnaire data for detailed analysis.

**FIGURE 9:**
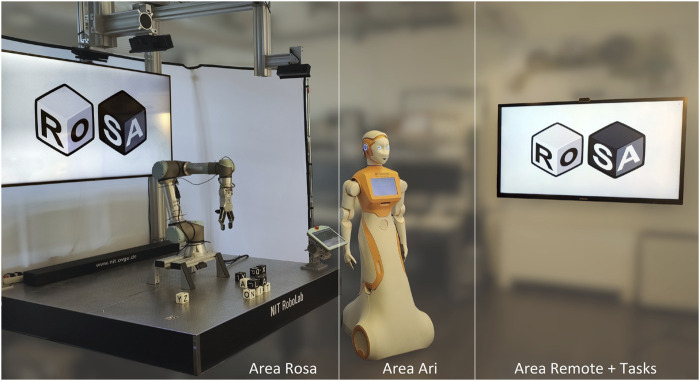
The local RoSA system setup with the three interaction areas (each with a touch screen) for the stationary robot *Rosa*, humanoid robot *Ari*, and a monitor for tasks or remote control.

The spatial arrangement of the robot interaction areas is illustrated in Figure 9. Following is the detailed procedure of the experiment.

### 4.2 Experiment procedure

**Declaration of consent** [*Table near monitor*]: Participants receive a detailed explanation of the study objectives, procedures, and potential risks involved. They are asked to provide their informed consent to participate voluntarily.

**Socio-demographic questionnaire** [*Monitor*]: At this stage, demographic and sociological data were collected, including participants’ age, gender, educational background, employment status, and any vision or hearing impairments. Additionally, participants reported their prior experience with collaborative robots, artificial intelligence, programming, and touchscreen use.

**Task set 1 (learning phase)** [*Rosa, Ari*]: Wake up and put *Rosa* and *Ari* to sleep using single modalities.

1. Wake up *Rosa* using speech
2. Sleep *Rosa* using touch
3. Wake up *Ari* using touch
4. Sleep *Ari* using speech

**Task set 2 (exploration phase)** [*Rosa, Ari*]: Personalization and playing a motion using any modality.

1. Change voice of *Rosa*
2. Change the eye color of *Ari*
3. Make *Ari* dance
4. Make *Rosa* nod

**Task set 3 (learning phase)** [*Rosa*]: Moving the cube using single modalities.

1. Build a small pyramid $\overset{□}{■\;■}$ using touch
2. Spell 
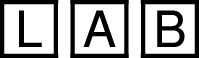
 using touch
3. Build a small pyramid $\overset{□}{■\;■}$ using speech
4. Spell 
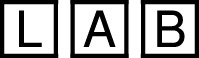
 using speech

**Task set 4 (exploration phase)** [*Rosa*]: Moving the cube using any modality.

1. Make *Rosa* give you a cube
2. Spell 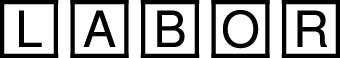

3. Build a big three layer pyramid 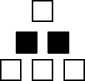

**Main questionnaire** [*Monitor*]: The questionnaire includes standardized assessments UMUX (Finstad, 2010), SUS (Brooke, 1996), ASQ (Lewis, 1991), PSSUQ (Lewis, 2002) and UEQ-S (Schrepp et al., 2017), which measure the user experience of the whole system. In addition, custom Likert-scale questions (7-point) were used to evaluate satisfaction with the interaction modules for each robot and for the system overall.

**Remote interaction + interview** [*Monitor*]: The remote segment of the experiment used the think-aloud method, in which participants engaged in open-ended exploration rather than following a specific task. This approach aimed to identify critical features and possible pressure points. An interview followed this exploration. To prevent bias, the remote phase commenced only after the on-site experiment had been thoroughly analyzed.

**FIGURE 10:**
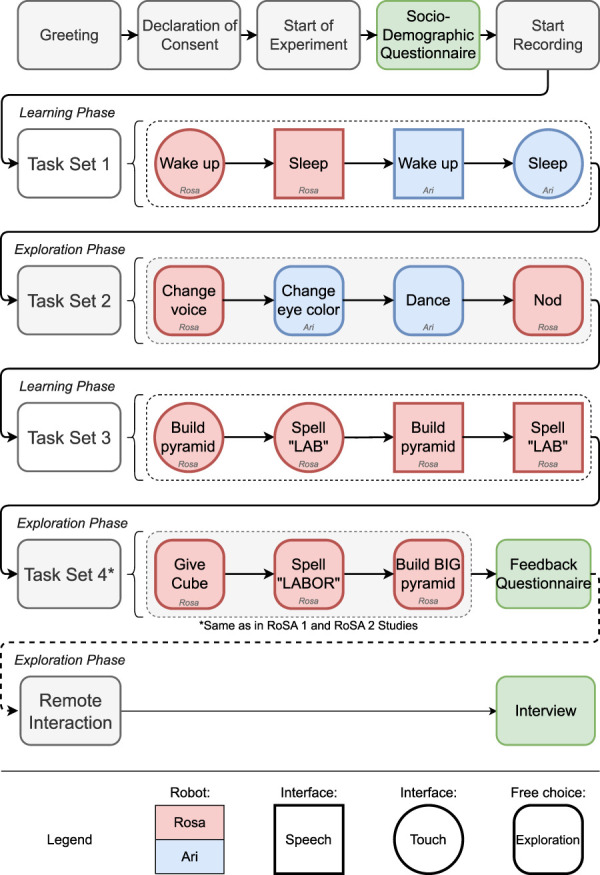
Experimental procedure: Tasks with robots *Rosa* and *Ari* are color-coded. Interaction modalities are indicated by shapes—circles for touch, squares for speech, and rounded squares for hybrid options.

The complete layout of the experiment procedure is summarized in Figure 10.

### 4.3 Participants

**FIGURE 11:**
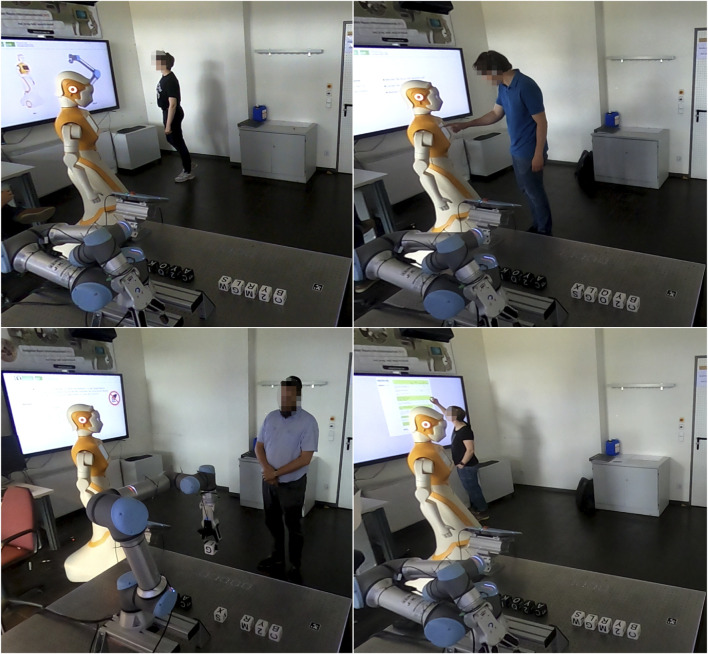
Experimental Study phases: Introduction (Top Left), Learning Phase (Top Right), Exploration Phase (Bottom Left), and Questionnaire (Bottom Right).

Figure 11 shows four participants during different phases of the experiment: Introduction (Declaration of consent), Learning Phase, Exploration Phase, and Questionnaire.

All 10 participants who completed the experiment had prior experience with touchscreens, while half of them also reported some experience with robots. The group consisted of five males and five females, aged between 20 and 40, with the majority falling within the 20 to 30 age range.

## 5 Results

This section presents the findings of the comparative study, focusing on the evaluation of touch and speech interaction modalities, as well as the overall performance of the RoSA 3 system. The results are based on task completion times, user preferences, questionnaire feedback, and manual observations.

### 5.1 Touch vs. speech: task time analysis

The time required to complete tasks using touch and speech modalities was analyzed. The tasks included spelling the word 
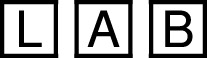
 with speech and touch modalities in Task Set 3 and completing the first three letters of the word 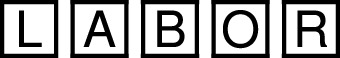 during the exploratory task in Task Set 4. Timing started when participants initiated the task and ended when the experimenter confirmed completion.

**FIGURE 12:**
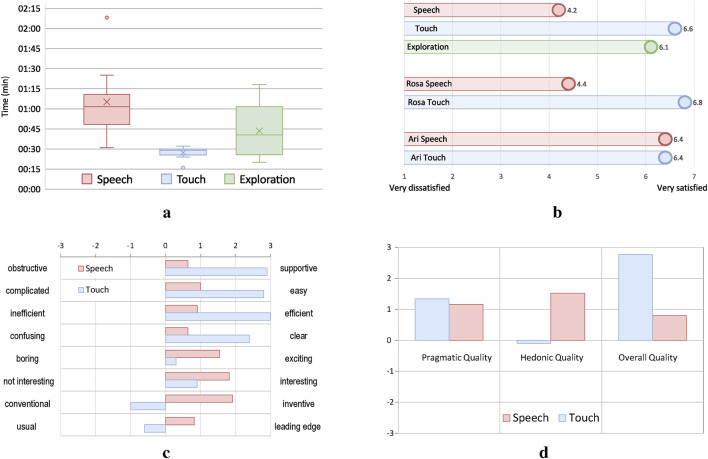
Summary of the experimental results for touch and speech modalities. **(a)** Average task completion times per modality. In the speech and touch tasks, users were restricted to one input type. The broader spread during the exploration phase suggests varied modality use, potentially including more frequent use of speech. **(b)** User satisfaction across tasks and modalities. Touch received the highest overall ratings. Speech and touch interfaces were also evaluated separately for *Rosa* and *Ari*, revealing slight differences in user preference between the robots. **(c)** UEQ-S item-level ratings for speech and touch interfaces across bipolar adjective pairs. Positive values indicate favorable user perception on each scale. **(d)** Hedonic vs. pragmatic quality of the interfaces. Pragmatic reflects task efficiency and clarity; hedonic captures user enjoyment and engagement.

The touchscreen interface had the shortest average task completion time at 27.8 s, followed by the exploration task at 43.5 s. The speech tasks required more time, with an average of 77.2 s. One participant required significantly longer using speech interface, due to manual correction of multiple misplaced blocks. Figure 12a summarizes the time results.

### 5.2 Touch vs. speech: user preferences and satisfaction

During the exploration tasks in Task Set 2, participants preferred speech for 90% of interactions with both robots. For cube manipulation in Task Set 4, participants initially preferred speech for 58% of the tasks. However, this preference decreased to 47% by the end of the task as some participants switched to touch.

Ratings for interaction modalities, displayed in Figure 12b, indicate that both modalities were rated equally for *Ari*. For *Rosa*, participants rated the touchscreen interface higher than the speech modality. Considering the overall, robot independent use, participants rated the touchscreen modality the highest followed by free exploration, followed by the speech modality.

### 5.3 Touch vs. speech: hedonic vs. pragmatic quality

The UEQ-S analysis assessed both pragmatic quality, i.e., the task-related aspects such as efficiency and clarity, and hedonic quality, which reflects user enjoyment and engagement. As shown in Figure 12d, the touchscreen interface scored higher in pragmatic dimensions, while speech was rated more positively in hedonic aspects. Overall quality scores (Figure 12c) align with the direct modality ratings shown in Figure 12b, supporting the observed trade-off between precision and naturalness across input types.

### 5.4 System evaluation: time analysis between all RoSA studies

Task completion times for RoSA 3 were compared with RoSA 1 and 2. Table 1 summarizes the results. RoSA 3 had the shortest average completion time (3:46 min), compared to 19:35 min for RoSA 1 and 25:20 min for RoSA 2.

**TABLE 1:** Comparison: Time efficiency.

Study	Task 1	Task 2	Task 3	Total
RoSA 1 (WoZ)	00:01:46	00:07:57	00:09:52	00:19:35
RoSA 2	00:01:21	00:12:56	00:11:06	00:25:20
**RoSA 3**	**00:00:27**	**00:01:22**	**00:01:57**	**00:03:46**

Bold values indicate the shortest (i.e., best) times for each task.

### 5.5 Speech command success-rate analysis

As the system uses Picovoice as a local SLU component, which directly maps audio signals to intents, this study focuses on the intent recognition accuracy rather than traditional word-level metrics. Simple intents such as *Help*, *Wake Up*, and *Sleep* were recognized reliably and triggered correctly in 95% of the cases during the experiment.

Looking at overall command success rates, 67.2% of the commands issued via speech input were executed successfully by the system, compared to 81.6% of the commands issued via touch input. Successful execution in this context means that the robot performed a *RobotAction*, such as manipulating a cube or changing its state, and confirmed completion with an “OK” message. A command was considered unsuccessful if the Commander returned an error and no *RobotAction* was performed. This includes commands issued during the initial learning phase as well as user errors due to logical issues, such as attempting to pick up a partially obstructed cube or stacking a cube onto itself. The discrepancy between speech and touch input reflects the higher likelihood of recognition or interpretation errors in the speech modality, particularly for more complex command structures.

### 5.6 System evaluation: questionnaire analysis

The user experience scores from the standardized questionnaires (UMUX, SUS, PSSUQ, and ASQ) were normalized to a scale of 0%–100% for consistency. The total average score across all questionnaires was calculated to be 75.58%, indicating a positive overall user experience with the system. RoSA 3 achieved the highest scores among the three system versions or came close to the highest score of the RoSA 1 system in the case of the UMUX questionnaire. The breakdown of scores by questionnaire type is shown in Table 2.

**TABLE 2:** Comparison of usability and user-experience questionnaires.

Metric	SUS	UMUX	PSSUQ	ASQ
Answer Range	1 to 5	1 to 7	1 to 7, NA	1 to 7, NA
Score Range	0 to 100	0 to 100	1 to 7	1 to 7
Nr. of Questions	10	4	16	3
Avg. Score	82.00	70.42	5.39	5.10
Std. Deviation	10.19	17.94	12.89	9.80
Normalized Score	**82.00**	**70.42**	**77.05**	**72.86**
Comparison with other RoSA studies				
RoSA 1 (WoZ)	79.24	**71.53**	73.70	71.60
RoSA 2	72.27	57.57	62.90	64.06
**RoSA 3 (this study)**	**82.00**	70.42	**77.05**	**72.86**

Bold values indicate the highest (i.e., best) scores.

### 5.7 Statistical analysis

A Pearson correlation analysis was conducted between all collected questionnaire items and objective performance measures from the experiment, including task completion times and the number of errors encountered across different input modalities. The goal was to explore potential relationships between subjective user evaluations and observed interaction performance. However, it is important to note that the statistical power of this analysis is limited due to the small sample size (n=10) . As such, the results should be interpreted with caution, and no claims about significance or generalizability are made.

• A negative correlation was observed between participant age and overall system satisfaction scores (r=−0.89) .

• Older participants tended to rate the system as more complex (r=0.66) and frustrating (r=0.86) compared to younger participants.

• Participants who rated the speech interface positively also gave higher overall system scores (r=0.67) .

• Conversely, participants who rated speech performance poorly for the *Rosa* robot often described the system as overly complex (r=−0.80) .

• Preferences for the touchscreen interface varied by robot type. Participants with less programming experience favored touchscreen interactions with humanoid robots (r=−0.58) , while those with more programming experience preferred touchscreen interactions with industrial robots (r=0.70) .

• The overall personal speech and touch modality evaluation contribute differently to the overall system score: speech has a higher impact on the overall system score (r=0.82) than touch (r=−0.06) .

### 5.8 Remote interview

In the remote interviews, participants commented that the commands and operations they had learned during on-site interaction were also usable in the remote environment. They noted that adapting to reduced information about the robot’s state took some getting used to. They emphasized the necessity of audio and video feeds, or possibly a 3D digital twin, combined with indicators such as progress bars to maintain a sense of control. Swiping between tabs was found to be unintuitive, suggesting that a learning phase for the touch interface or additional guidance could be beneficial. Participants also highlighted the need for the remote interface to access manufacturer screens for low-level or administrative tasks, as well as the importance of autostart and reboot options. On the positive side, the uniformity and familiarity of the web-based interface across different locations was well-received.

### 5.9 Summarized results

The touchscreen interface proved more efficient, particularly in tasks requiring precision, while speech excelled in natural, hands-free interaction during exploratory tasks. Touch was rated higher in pragmatic quality for its reliability and task efficiency, whereas speech was valued for its hedonic quality and engaging nature. Participants preferences shifted based on task demands, favoring speech initially but transitioning to touch for accuracy. The RoSA 3 system outperformed its predecessors in task completion times and user satisfaction, although speech interactions faced challenges like recognition errors, leading to a reliance on touch as a fallback.

## 6 Evaluation and discussion

This study evaluated the effectiveness of touch and speech interaction modalities within the RoSA 3 framework, highlighting their respective strengths and limitations in Human-Robot Interaction (HRI) and telerobotics. The findings underscore the importance of task-specific modality selection and provide insights into how multimodal systems can enhance user experience.

### 6.1 Time efficiency

The touchscreen interface consistently outperformed speech in task completion times, particularly in precise, task-oriented scenarios like cube manipulation. This advantage can be attributed to the direct and deterministic nature of touch inputs, which provide immediate feedback and eliminate ambiguities inherent in speech-based interaction. Speech, while slower, offered flexibility and a natural interaction flow, making it preferable for exploratory tasks or when participants sought hands-free operation.

A significant reduction in completion time was also influenced by the structured learning phase introduced in the RoSA 3 system. This phase familiarized participants with the system before task execution, a benefit not provided in earlier studies with RoSA 1 and 2, where learning was integrated into task time. Moreover, this approach reflects a more realistic scenario for future industrial deployments, as workers are typically trained in advance to operate such systems efficiently in their professional environment.

Additionally, participants were not instructed to prioritize speed, allowing them to interact naturally. Some deliberately took extra time to explore the system, which may have influenced overall completion times but provided valuable insights into user behavior and interaction preferences.

While the raw time data can be analyzed as a quantitative measure of efficiency, a qualitative efficiency comparison between the studies is challenging due to differences in system capabilities, and participant familiarity with the modalities. The studies also show the importance of a structured learning phase to familiarize participants with the system, which can significantly impact task completion times and overall user experience.

### 6.2 User preferences and quality dynamics

During the exploratory phases, participants initially favored speech due to its intuitive and natural interaction flow, which they found engaging and enjoyable (hedonic quality). This can be seen particularly well in Figure 12b, where satisfaction with the speech interface varies significantly between the humanoid robot *Ari* and the industrial robot *Rosa*. While *Ari’s* speech interface was rated similarly to its touch interface, *Rosa’s* speech interface was evaluated considerably lower.

Although the humanoid appearance of *Ari* may have had a minor influence, we attribute the difference primarily to the varying complexity of the speech interfaces. Interaction with *Ari* was limited to simple option selections, whereas controlling the *Rosa* robot via speech required more complex, multistep interaction, such as specifying cube positions or coordinating actions—making errors more likely and the experience more frustrating.

However, when tasks required more precision, speech recognition errors and additional confirmations made speech input less appealing. In these cases, the touchscreen’s efficiency, reliability, and direct control (pragmatic quality) became more attractive. By the end of the study, most participants preferred touch for cube-related tasks. This duality highlights the complementary strengths of both modalities: speech offers a more human-like, fluid interaction, while touch excels in precise, task-oriented operations.

Because the same input modalities are used both on-site and remotely, these findings also transfer directly to telerobotics. Participants noted that, to better understand the robot’s status and ongoing tasks, a live audio and video feed would be beneficial. The system’s design facilitates seamless operation in both local and remote scenarios, making it suitable for supervisory tasks in environments with varying latency or network quality. Consequently, this flexible approach paves the way for a new working style, where operators can switch between local and remote operations as needed, showing particular promise for long-duration tasks that benefit from a hybrid work mode.

### 6.3 Evaluating the systems through the lens of multimodality

Reflecting on the evolution of RoSA systems, the progression in multimodal interaction capabilities has been a defining feature of its development. The preceding version RoSA 2 demonstrated functional multimodal interactions but were limited by racing conditions and conflicts arising from simultaneous inputs, which can be summarized as “Sequential Multimodality”. These limitations highlighted the need for more advanced input management strategies to achieve a seamless user experience.

RoSA 3 eliminated the racing conditions by introducing a new “Buffered Multimodality” approach. This system progressively gathers input from multiple modalities before executing commands, ensuring context-aware and conflict-free interactions. While this method introduces slight delays, it marks a clear departure from the challenges of earlier versions, prioritizing reliability.

**FIGURE 13:**
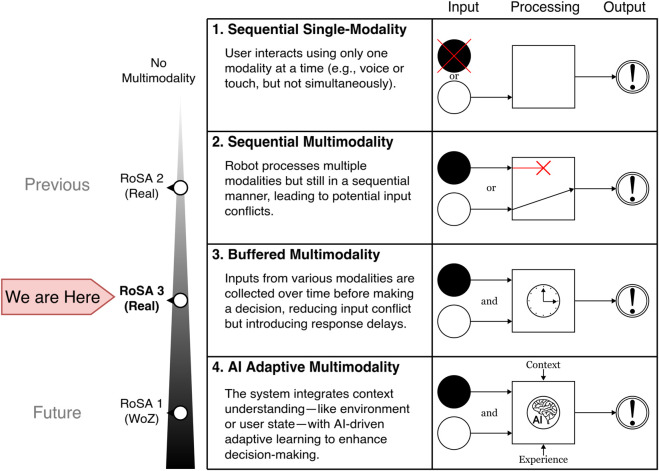
Levels of MultiModality and current RoSA implementations.

RoSA 1, by being a Wizard of Oz study, achieved the highest level of multimodality so far. The human operator (Wizard) was able to interpret user inputs contextually, leveraging implicit learning and adaptation throughout the experiment. This created the illusion of “AI-adaptive Multimodality”, where the system appeared to learn and adapt to user preferences and behaviors. The aspirational goal for future iterations could be to achieve true AI-adaptive multimodality. In such a system, the robot would dynamically understand context, learn from user behavior, and adapt its responses in real-time, offering a seamless and intuitive interaction experience. Figure 13 illustrates this progression, emphasizing the increasing sophistication of the RoSA systems in managing and integrating multiple input modalities effectively.

### 6.4 Limitations and future improvements

While the presented study offers insights into hybrid human-robot interaction using touch and speech modalities, several limitations should be acknowledged to contextualize the findings.

The study was conducted with a small sample of ten participants. This limited size constrains the generalizability of the results. The primary aim was to explore interaction patterns and modality trade-offs in depth, as part of a continued research line across three RoSA system iterations. Future work will incorporate larger and more diverse user groups to improve statistical power and external validity.

While remote interaction was a partial focus, the study did not include detailed latency measurements. The system was evaluated from a user experience perspective, and although network conditions were stable enough not to interfere with task execution, future studies will integrate performance logging and analysis of recognition errors.

The use of ROS 1 (Noetic) instead of ROS 2 was driven by hardware constraints. Specifically, the humanoid robot *Ari* currently only supports ROS 1. Although some system components are being prepared for ROS 2 migration, full transition was not feasible within the scope of this study.

By using PicoVoice as the NLU component, a resource-efficient implementation could be realized. PicoVoice maps user intents directly from the speech signal, which works particularly well for short commands and statements, however, this approach limits dialogue complexity and restricts system-level analysis. Specifically, it is only possible to determine whether an utterance was successfully mapped to an intent, without deeper insights. It is not possible to analyse whether specific parts of the utterance may have caused recognition issues.

In terms of user population, most participants had prior exposure to touch-based technology, and some had experience with robots, which may influence the interaction preferences observed. Additionally, task scenarios focused primarily on cube manipulation, limiting the range of real-world application scenarios. Expanding the system to include collaborative assembly or diagnostic tasks is planned for future iterations.

To maintain comparability with previous RoSA studies, we retained the same set of standardized questionnaires (UMUX, SUS, PSSUQ, ASQ). Although some of these instruments partially overlap in focus, they were intentionally selected for continuity and because their limited length imposed minimal additional workload on participants. Short formats like ASQ (3 items) and UMUX (4 items) allowed us to assess both task-specific and overall usability efficiently. While more recent tools such as UMUX-Lite may offer similar insights with even fewer items, we prioritized consistency in this iteration and plan to revise the questionnaire set and include UMUX-Lite in future studies.

## 7 Conclusion

This work has shown that both touch and speech can successfully bridge local and remote interaction scenarios without substantial changes to the underlying interface. Such hybrid usability paves the way for future industrial deployments, where operators will seamlessly switch between on-site and remote control depending on task demands.

While speech encountered issues in precision-heavy tasks, ongoing advances in large language models (LLMs) and reasoning agents hold promise for more natural, open-ended dialogue and improved contextual understanding (Wei et al., 2022; Mialon et al., 2023). By equipping the Commander module with LLM-based reasoning (Yao et al., 2023; Schick et al., 2024), the system could dynamically select the best action or modality based on learned experience and contextual cues, moving toward genuinely adaptive multimodality. Recent work integrating LLMs into embodied robotic scenarios (Ichter et al., 2023; Driess et al., 2023) also underscores the potential for AI-driven tools that bridge the gap between human instructions and robotic execution in both local and remote environments.

A structured learning phase proved crucial, underscoring the practical importance of training users before complex operations. In real-world industrial environments, familiarizing workers with a system’s capabilities can lead to significant time savings and smoother workflows. Though users often gravitate toward their familiar methods, the ability of a system to integrate novel modalities without sacrificing reliability or performance will be key to long-term acceptance.

Moving forward, a hybrid local and remote HRI marks a step toward cooperative robots that adapt their interaction style to the needs and preferences of the users, whether working side-by-side on the factory floor or assisting remotely from afar.

## Data Availability

The raw data supporting the conclusions of this article will be made available by the authors, without undue reservation.

## Footnotes

1 see https://picovoice.ai/

## References

[B1] Abdelrahman A. A. Strazdas D. Khalifa A. Hintz J. Hempel T. Al-Hamadi A. (2022). Multimodal engagement prediction in multiperson human–robot interaction. IEEE Access 10, 61980–61991. 10.1109/access.2022.3182469

[B2] Afanasyev I. Mazzara M. Chakraborty S. Zhuchkov N. Maksatbek A. Yesildirek A. (2019). “Towards the internet of robotic things: analysis, architecture, components and challenges,” in 2019 12th international conference on developments in eSystems engineering (DeSE), 3–8. 10.1109/DeSE.2019.00011

[B3] Angelini L. Baumgartner J. Carrino F. Carrino S. Caon M. Khaled O. A. (2016). “Comparing gesture, speech and touch interaction modalities for in-vehicle infotainment systems,” in Proceedings of the 28ième conférence francophone sur l’Interaction Homme-Machine, 188–196. 10.1145/3004107.3004118

[B4] Bongiovanni A. De Luca A. Gava L. Grassi L. Lagomarsino M. Lapolla M. (2022). “Gestural and touchscreen interaction for human-robot collaboration: a comparative study,” in International conference on intelligent autonomous systems (Springer), 122–138.

[B6] Brooke J. (1996). “ *SUS: a “Quick and dirty” usability scale*. Tech. Rep., digital equipment corporation, reading, ma,” in Published in usability evaluation in industry. Editors Jordan P. W. Thomas B. Weerdmeester B. A. McClelland I. L. (London: Taylor & Francis).

[B5] Driess D. Xia F. Sajjadi M. S. M Lynch C. Chowdhery A. Aakanksha I. (2023). PaLM-E: an embodied multimodal language model. Proceedings of the 40th International Conference on Machine Learning, Honolulu, Hawaii. 10.5555/3618408.3618748

[B7] Finstad K. (2010). The usability metric for user experience. Interact. Comput. 22, 323–327. 10.1016/j.intcom.2010.04.004

[B8] Hempel T. Al-Hamadi A. (2023). “On contextual perception of workers in complex production environments,” in Engineering for a changing world: proceedings of the 60th ilmenau scientific colloquium (Ilmenau, Germany: Technische Universität Ilmenau), 8. 10.22032/dbt.58931

[B9] Hentout A. Mustapha A. Maoudj A. Akli I. (2019). Human–robot interaction in industrial collaborative robotics: a literature review of the decade 2008–2017. Adv. Robot. 33, 764–799. 10.1080/01691864.2019.1636714

[B10] Ichter B. Brohan A. Chebotar Y. Finn C. Hausman K. Herzog A. (2023). “Do as i can, not as i say: grounding language in robotic affordances,” in Proceedings of the 6th conference on robot learning. Editors Liu K. Kulic D. Ichnowski J., 287–318.

[B11] Kapić Z. Crnkić A. Mujčić E. Hamzabegovic J. (2021). A web application for remote control of ros robot based on websocket protocol and django development environment. IOP Conf. Ser. Mater. Sci. Eng. 1208, 012035. 10.1088/1757-899X/1208/1/012035

[B12] Keshvarparast A. Battini D. Battaia O. Pirayesh A. (2024). Collaborative robots in manufacturing and assembly systems: literature review and future research agenda. J. Intelligent Manuf. 35, 2065–2118. 10.1007/s10845-023-02137-w

[B13] Lewis J. R. (1991). Psychometric evaluation of an after-scenario questionnaire for computer usability studies: the asq. ACM SIGCHI Bull. 23, 78–81. 10.1145/122672.122692

[B14] Lewis J. R. (2002). Psychometric evaluation of the post-study system usability questionnaire: the pssuq. Proc. Hum. Factors Ergonomics Soc. Annu. Meet. 36, 1259–1260. 10.1177/154193129203601617

[B15] McTear M. (2022). Conversational ai: dialogue systems, conversational agents, and chatbots. Springer Nature.

[B16] Mialon G. Dessì R. Lomeli M. Nalmpantis C. Pasunuru R. Raileanu R. (2023). Augmented Language models: a survey. arXiv:2302.07842 Preprint

[B17] Morgan A. A. Abdi J. Syed M. A. Q. Kohen G. E. Barlow P. Vizcaychipi M. P. (2022). Robots in healthcare: a scoping review. Curr. Robot. Rep. 3, 271–280. 10.1007/s43154-022-00095-4 36311256 PMC9589563

[B18] Profanter S. Perzylo A. Somani N. Rickert M. Knoll A. (2015). “Analysis and semantic modeling of modality preferences in industrial human-robot interaction,” in 2015 IEEE/RSJ international conference on intelligent robots and systems (IROS), 1812–1818. 10.1109/IROS.2015.7353613

[B19] Radfar M. Mouchtaris A. Kunzmann S. Rastrow A. (2021). Fans: fusing asr and nlu for on-device slu. arXiv, 1224–1228. 10.21437/interspeech.2021-793

[B20] Romeo L. Petitti A. Marani R. Milella A. (2020). Internet of robotic things in smart domains: applications and challenges. Sensors Basel, Switz. 20, 3355. 10.3390/s20123355 PMC7349752 32545700

[B21] Sadiku M. N. O. Ajayi-Majebi A. J. Adebo P. O. (2023). “Robotic automation in manufacturing,” in Explores the use of robotic automation, including telerobotics, in manufacturing processes for improved safety and efficiency (Cham: Springer). 10.1007/978-3-031-23156-8/-3

[B22] Schick T. Dwivedi-Yu J. Dessì R. Raileanu R. Lomeli M. Hambro E. (2024). Toolformer: language models can teach themselves to use tools. Adv. Neural Inf. Process. Syst. 36, 68539–68551. 10.48550/arXiv.2302.04761

[B23] Schmaus P. Leidner D. Krüger T. Bayer R. Pleintinger B. Schiele A. (2020). Knowledge driven orbit-to-ground teleoperation of a robot coworker. IEEE Robotics Automation Lett. 5, 143–150. 10.1109/LRA.2019.2948128

[B24] Schrepp M. Hinderks A. Thomaschewski J. (2017). Design and evaluation of a short version of the user experience questionnaire (ueq-s). Int. J. Interact. Multimedia Artif. Intell. 4, 103–108. 10.9781/ijimai.2017.09.001

[B25] Senft E. Hagenow M. Welsh K. Radwin R. Zinn M. Gleicher M. (2021). Task-level authoring for remote robot teleoperation. Front. Robotics AI 8, 707149. 10.3389/frobt.2021.707149 PMC8502825 34646866

[B26] Stefanuto B. Piardi L. Júnior A. O. Vallim M. Leitão P. (2023). “Remote lab of robotic manipulators through an open access ros-based platform,” in 21st IEEE international conference on industrial informatics, INDIN 2023, lemgo, Germany, july 18-20, 2023 (IEEE), 1–6. 10.1109/INDIN51400.2023.10218202

[B27] Strazdas D. Hintz J. Felßberg A.-M. Al-Hamadi A. (2020). Robots and wizards: an investigation into natural human–robot interaction. IEEE Access 8, 207635–207642. 10.1109/ACCESS.2020.3037724

[B28] Strazdas D. Hintz J. Khalifa A. Abdelrahman A. A. Hempel T. Al-Hamadi A. (2022). Robot system assistant (rosa): towards intuitive multi-modal and multi-device human-robot interaction. Sensors 22, 923. 10.3390/s22030923 35161671 PMC8838571

[B29] Su H. Qi W. Chen J. Yang C. Sandoval J. Laribi M. A. (2023). Recent advancements in multimodal human–robot interaction. Front. Neurorobotics 17, 1084000. 10.3389/fnbot.2023.1084000 PMC10210148 37250671

[B30] Wei J. Bosma M. Zhao V. Y. Guu K. Yu A. W. Lester B. (2022). Finetuned Language Models Are Zero Shot Learners. International Conference on Learning Representations (ICLR) 35. 10.48550/arXiv.2109.01652

[B31] Yao S. Zhao S. Yu T. Du N. Shi S. Cui L. (2023). ReAct: synergizing reasoning and acting in language models. arXiv:2210.03629 Preprint

